# Exploring biases related to the use of large language models in a multilingual depression corpus

**DOI:** 10.1038/s41598-025-19980-x

**Published:** 2025-10-16

**Authors:** Paula Andrea Perez-Toro, Judith Dineley, Raquel Iniesta, Yuezhou Zhang, Faith Matcham, Sara Siddi, Femke Lamers, Josep Maria Haro, Brenda W. J. H. Penninx, Amos A. Folarin, Tomas Arias-Vergara, Juan Rafael Orozco-Arroyave, Elmar Nöth, Andreas Maier, Til Wykes, Srinivasan Vairavan, Richard Dobson, Vaibhav A. Narayan, Matthew Hotopf, Nicholas Cummins

**Affiliations:** 1 Institute of Psychiatry, Psychology & Neuroscience, King’s College London, London, UK; 2 Pattern Recognition Lab, Friedrich-Alexander-Universität Erlangen-Nürnberg, Erlangen, Germany; 3 GITA Lab, Faculty of Engineering. Universidad de Antioquia, Medellín, Colombia; 4 School of Psychology, University of Sussex, Falmer, UK; 5 Parc Sanitari Sant Joan de Deu, Fundació Sant Joan de Deu, CIBERSAM, Universitat de Barcelona, Barcelona, Spain; 6 Department of Psychiatry, Amsterdam Public Health Research Institute and Amsterdam Neuroscience, Amsterdam, the Netherlands; 7 NIHR Biomedical Research Centre at South London, Maudsley NHS Foundation Trust, King’s College London, London, UK; 8 Janssen Research and Development LLC, Titusville, NJ USA; 9 Institute of Health Informatics, University College London, London, UK; 10 Davos Alzheimer’s Collaborative, Philadelphia, USA; 11 Thymia, London, UK

**Keywords:** Depression, Large language models, Biases in AI, Text analysis, Applied mathematics, Computational science

## Abstract

Recent advancements in Large Language Models (LLMs) present promising opportunities for applying these technologies to aid the detection and monitoring of Major Depressive Disorder. However, demographic biases in LLMs may present challenges in the extraction of key information, where concerns persist about whether these models perform equally well across diverse populations. This study investigates how demographic factors, specifically age and gender affect the performance of LLMs in classifying depression symptom severity across multilingual datasets. By systematically balancing and evaluating datasets in English, Spanish, and Dutch, we aim to uncover performance disparities linked to demographic representation and linguistic diversity. The findings from this work can directly inform the design and deployment of more equitable LLM-based screening systems. Gender had varying effects across models, whereas age consistently produced more pronounced differences in performance. Additionally, model accuracy varied noticeably across languages. This study emphasizes the need to incorporate demographic-aware models in health-related analyses. It raises awareness of the biases that may affect their application in mental health and suggests further research on methods to mitigate these biases and enhance model generalization.

## Introduction

There is a steadily increasing interest in using Large Language Models (LLMs) in clinical settings, as these approaches represent advanced iterations in the field of Natural Language Processing (NLP)^1,2^. These models use extensive training on diverse datasets to generate coherent, context-aware outputs. Despite recent technological advances, biases in LLMs remain a considerable concern^3–5^. Biases occur when the outputs disproportionately favour or disadvantage individuals based on characteristics such as race, gender, age, or socioeconomic status. As data used to train LLMs reflects existing human prejudices, they can inadvertently lead to biased model outputs. Biases in LLMs can manifest as stereotypes or as an unintentional favouring of certain demographic groups^4^. Moreover, most existing work focuses on monolingual English data and overlooks how LLMs perform in multilingual and demographically diverse contexts, raising risks of biased or inaccurate predictions in real-world deployments .

Applying LLMs in mental health presents unique opportunities and challenges, particularly concerning biases. LLMs can be employed to support mental health assessments, provide therapeutic communication, or help in crisis intervention by analyzing speech or text patterns indicative of mental health issues^6,7^. The link between alterations spoken language and mental health is especially clear in Major Depressive Disorder (MDD)^8^. MDD affects millions worldwide. A 2018 Organisation for Economic Co-operation and Development (OECD) report notes that depression impacted about 21 million people within the European Union by 2016^9,10^. In particular, its manifestation and prevalence vary by demographics, with about 5% of adults (approximately 6% of women and 4% of men) suffering from depression and an even higher rate (5.7%) observed in adults over 60 years old^11^. Recent years have seen a notable rise in research applying LLMs to mental health. In a 2025 scoping review, 71% of studies using LLMs in mental health focused on screening or detecting disorders, with depression being the most common target ($\approx$35%)^7^. These models leverage transformers’ ability to understand context and subtle language patterns; compared to earlier machine-learning or human assessments, LLM-based approaches show advantages in information processing and consistency^7^. Among mental health conditions,is particularly amenable to language-based modeling, as it is known to alter both the content and style of communication^8,12^, depressed individuals often use negative language, focusing on themes of sadness and using absolutist terms, which can reflect a polarized worldview^13^. LLMs have the potential to assist in utilising these linguistic cues to develop effective models for detecting mental health changes. Recent studies on non-clinical social media data, such as Reddit or Twitter, report high LLM classification performance for depression detection, which often achieve F1-scores between 0.85 and 0.96 on fine-tuned models, likely due to the availability of abundant, typed user-generated content^14^. In contrast, performance on elicited speech transcripts remains more modest, with zero-shot LLMs achieving F1-scores in the range of 0.50 to 0.73, likely due to the noisier input from ASR, linguistic variability, and smaller dataset sizes^15,16^.

However, critical challenges remain before such systems can be reliably used in real-world diagnostics. Most trained LLMs, developed from extensive text datasets, often inherit biases ranging from gender to cultural insensitivities, which can negatively impact their application in mental health settings; e.g., when aiding the monitoring of MDD symptoms^17–20^. This is especially troubling in mental health, where factors like age, gender, socioeconomic status, and cultural background influence language use and symptom expression. If these biases are not addressed, LLMs may misinterpret symptom-related cues across different populations ^21^. For instance, a model might fail to detect expressions of distress in non-standard dialects or misclassify culturally specific emotional language. Such misinterpretations can lead to inadequate interventions, potentially worsening disparities in mental health care. Several studies have highlighted the importance of demographic considerations in supporting mental health diagnostics and monitoring through these models. Recent research emphasizes the need for demographic-aware models in health-related text analyses, warning that ignoring demographic factors can lead to biased and inaccurate predictions^3,22^ and limit the effectiveness of LLMs in mental health applications^5^. For example, LLMs often stereotype women as nurses or assistants and men as doctors or engineers, reinforcing male-dominated roles in leadership and technical fields^23^. These biases can have serious real-world consequences, with models potentially leading to incorrect diagnoses, inappropriate treatments, and increased risk of adverse events, such as suicide in MDD cases^24^. Addressing such biases is critical to minimizing the risk of discrimination and ensuring that LLMs produce fairer/equitable outcomes for diverse populations.

To address persistent concerns about fairness and performance disparities in multilingual mental health applications, this study focuses on investigating how demographic balancing (age and gender) affects the performance of state-of-the-art LLMs in classifying the severity of MDD symptoms across English, Spanish, and Dutch speech transcripts^25,26^. By evaluating models on carefully balanced and multilingual datasets, we aim to uncover where demographic and linguistic biases impact model predictions. We performed a systematical evaluation of the language generalization potential of several LLMs, offering empirical evidence of the performance variances when these models are applied to the different datasets. Thus, this study investigates three key research questions: (1) How do demographic factors such as age and gender influence the performance of LLMs in classifying depression symptom severity? (2) Do multilingual LLMs maintain consistent accuracy across different languages? (3) How does model architecture impact susceptibility to demographic and linguistic biases? In response to these questions, our key contributions are: (i) presenting an analysis of the effects of demographic (age/gender) balancing on severity symptom prediction in MDD performance, (ii) providing evidence of biases in different pre-trained LLMs related to age and gender, (iii) to the best of the authors knowledge, presenting one of the first studies on performing multilingual classification of depression using LLMs. Our findings provide practical guidance for the development of more equitable LLM-based diagnostic tools. They highlight the importance of representative data sampling and suggest that demographic-aware evaluation protocols can mitigate model bias, an essential step toward improving the fairness and reliability of LLMs in real-world mental health screening applications.

## Data

Our data is from the Remote Assessment of Disease and Relapse in Major Depressive Disorder (RADAR-MDD^25^) longitudinal cohort study examining the utility of multi-parametric remote measurement technologies (RMT), including speech, to measure changes in symptoms and predict relapse in people with MDD. The full eligibility and exclusion criteria for this study are published in^25^. The study had three recruitment sites: London, UK; Amsterdam, The Netherlands; and Barcelona, Spain. Ethical approval for these sites was obtained locally.

### Patient involvement

The experimental protocol was co-developed with a patient advisory board who shared their opinions on several user-facing aspects of the study, including the choice and frequency of survey measures, the usability of the study app, participant-facing documents, selection of optimal participation incentives, selection, and deployment of wearable device as well as the data analysis plan. The speech tasks and subsequent analysis were discussed specifically with this board.

### Ethics approval and consent to participate

Ethical approvals for study conduct were obtained from the Camberwell St Giles Research Ethics Committee (REC reference: 17/LO/1154), in London from the CEIC Fundacio Sant Joan de Deu (CI: PIC-128-17) in Barcelona, and from the Medische Ethische Toetsingscommissie VUms (METc VUmc registratienummer: 2018.012 – NL63557.029.17) in the Netherlands. RADAR-MDD was conducted per the Declaration of Helsinki and Good Clinical Practice, adhering to principles outlined in the NHS Research Governance Framework for Health and Social Care (2nd edition). All participants provided their informed consent to participate.

### Speech collection

For full details on the preparation and organization of the speech data, the interested reader is referred to^26^. In this work, we used the *free-response* data only to extract language features. In that task, participants were asked to speak about something they were looking forward to in the next seven days. They also completed an 8-item Patient Health Questionnaire (PHQ-8) scale to assess the severity of depression^27^. For our analysis the data was grouped into two partitions: (i) speech recordings associated with low symptom severity (PHQ-8<10); and (ii) speech recordings associated with high symptom severity (PHQ-8$\ge$10). Transcriptions were automatically generated using Whisper’s^28^ large model (see https://huggingface.co/openai/whisper-large-v3). This model has demonstrated strong multilingual transcription performance on benchmark datasets, with reported Word Error Rates (WER) of approximately 2.6% for English^28^, 5.3% for Spanish (fine-tuned)^29^, and around 4–5% for Dutch^30^ in clean, read-speech conditions.

The average number of words per participant and language were: English participants averaged 33.6±32.0, Spanish participants 28.4±32.2, and Dutch participants 34.6±20.9. The total number of samples and information on the distribution of the text files used in our analysis are presented in Tab.1.

**Table 1:** Demographic and clinical information of the participants for each dataset.

Group	Gender [W/M]	Age	PHQ-8
*English*			
High symptom severity	1193/360	44.71 (15.00)	15.22 (3.78)
Low symptom severity	1025/312	50.84 (15.22)	4.75 (2.75)
*Spanish*			
High symptom severity	319/140	51.54 (10.08)	16.28 (4.17)
Low symptom severity	192/161	52.68 (11.35)	5.14 (2.88)
*Dutch*			
High symptom severity	308/108	40.00 (16.78)	14.61 (3.74)
Low symptom severity	297/107	48.18 (16.43)	5.41 (2.63)

Values are expressed as mean (standard deviation). W: Women. M: Men. Age is given in years.

## Methods

Our study is based on the “word-embedding” paradigm, which is designed to capture relationships between words and the contextual variations of language usage^31^. LLMs-based methods have been extensively used for MDD detection. Models such as FlanT5^1,2^, BERT^32,33^, RoBERTa^34,35^, GPT^2,36^, and LLaMA^7,37^ have demonstrated promising potential in identifying MDD through language patterns. However, biases often arise due to the datasets on which these models are trained^21^. We selected a diverse set of LLMs based on architectural variety, encoder-only (e.g., BERT, RoBERTa, MentalBERT), decoder-only (e.g., GPT-2, LLaMA, MentaLLaMA), and encoder-decoder (FlanT5), as well as their multilingual coverage and relevance to mental health domains. A summary of the considered LLMs in this study can be found in Tab.2.

**Table 2:** Summary of the key properties of the Large Language Model utilized in this work.

LLM	Training Data	Based Architecture	Languages
FlanT5	Muffin^38^, T0-SF, Chain-of-Thought (CoT)^39^ and Natural Instructions v2^40,41^	Encoder/Decoder	Multilingual (41 languages)
RoBERTa	CommonCrawl^42^	Encoder	Multilingual (100 languages)
mBERT	Mental health-related posts collected from Reddit^43^	Encoder	English
GPT-2	WebText^18^	Decoder	English
BERT	Wikicorpus dataset^44^	Encoder	Multilingual (104 languages)
LLaMA	Two trillion tokens of data sourced from internet^45^	Decoder	Mainly English
MentaLLaMA	Interpretable Mental Health Instructions (IMHI)^37^	Decoder	English

### Bidirectional encoder representations from transformers (BERT)

Unlike conventional models that process words either in isolation or in sequential order, this approach uses the encoder part of the Transformer architecture^46^, which enables the model to process words in relation to all other words in a sentence. BERT learns multiple attention mechanisms, called heads, which operate in parallel to one another. It was trained using unlabelled text, with a training paradigm employing two key unsupervised learning tasks: (1) Masked Language Modeling (MLM), which involves predicting words that have been intentionally masked in a sentence; and (2) Next Sentence Prediction (NSP), trains the model to determine whether two given sentences logically follow each other, which enhances its ability to capture relationships across sentences. The model considered in this study has been pre-trained on the Wikicorpus^44^ dataset (see https://huggingface.co/google-bert/bert-base-multilingual-uncased). For classification tasks such as ours, it is common to compute an average of the word embeddings from the last layer, which consists of 768 units, to represent the input text^31^. Additionally, we considered using mentalBERT (mBert), which is pretrained in a non-clinical corpus collected from Reddit^43^, specifically targeted mental health-related discussions.

### A robustly optimized BERT pretraining approach (RoBERTa)

RoBERTa is based on the same principles of BERT, but offers improvements in the training process and model performance^47^. A key innovation in RoBERTa is the removal of the NSP task. Instead, it focuses only on the MLM task, introducing a novel aspect of dynamic masking. The masked words are changed dynamically across different training epochs, which prevents the model from memorizing the answers, the idea being to encourage a deeper learning of context and language structure. RoBERTa also employs multi-head attention to capture relationships between words and larger batch sizes during training, which has been shown to contribute to better model performance^47^. We utilise the RoBERTA-Base model, which was trained with filtered CommonCrawl data from 100 languages^42^. We average the embeddings before passing them to a classification layer.

### Generative pretrained transformer 2 (GPT-2)

This language model primarily focuses on text-generation tasks^18^. GPT-2 learns to predict the next word in a sequence by analyzing the preceding words. It utilises the MLM methodology to generate coherent text based on a given prompt. Unlike systems that use both the encoder and decoder of the Transformer, this model exclusively uses the decoder. GPT-2 was pretrained on WebText, an extensive dataset extracted from the Internet that includes 45 million links^18^. This dataset spans a wide range of topics and languages, enabling GPT-2 to perform a variety of text-based tasks without task-specific training. Its capabilities include translation, question answering, and content creation. GPT-2 also employs a multi-head attention mechanism, enabling it to effectively focus on different parts of the input sentence to predict the next word. In this study, we utilise the model GPT-2 Medium, with an output vector size of 1024 dimensions. Note that subsequent versions of GPT were not employed in this study due to privacy considerations, as using these versions requires training on OpenAI’s cloud.

### Text-to-text transfer transformer (T5)

The T5 model redefines the strategy for managing several NLP tasks by framing all text processing activities as a text-to-text problem^17^. Whether translating languages, summarizing articles, or answering questions, T5 processes the input text. It generates output text, thereby simplifying the application of machine learning models to a wide range of text-based tasks. This model uses a modified version of the Transformers architecture (both encoder and decoder), which introduces a unified approach by using MLM pretraining on a large corpus of text drawn from diverse sources^17^, followed by fine-tuning on specific tasks. This method allows T5 to rely on the insights gained from pretraining across multiple domains, making it suitable for a wide range of tasks with minimal task-specific adaptations. We utilise the fine-tuning language model T5 (FlanT5), trained on a corpus comprising texts from 41 languages. For classification tasks addressed in this work, we considered the model’s last hidden layer from the encoder part (1024 output dimension).

### Large language model meta AI (LLaMA)

LLaMA is a high-performance language model developed by Meta AI, optimized for efficiency and scalability^45^. Unlike traditional Transformer-based architectures that often require vast computational resources and parameter counts, LLaMA aims to optimize both model size and training strategy to deliver strong performance across a variety of natural language processing tasks, even under constrained computational conditions. Architectural improvements such as RMSNorm, SwiGLU activations, and rotary positional embeddings enhance training stability and performance. Trained with a causal language modeling objective, LLaMA achieves strong results even with limited labeled data. The [SPSVERBc1SPS] model used in this study is a decoder-only Transformer trained on 2 trillion tokens from licensed, public, and Internet-curated sources, covering a broad range of domains. In addition, we utilise MentaLLaMA (mLLaMA)^37^, which was trained with interpretable mental health instruction data specifically curated to improve performance on mental health-related tasks. Finally, we average the embeddings before passing them to a classification layer.

## Results

**Fig. 1:**
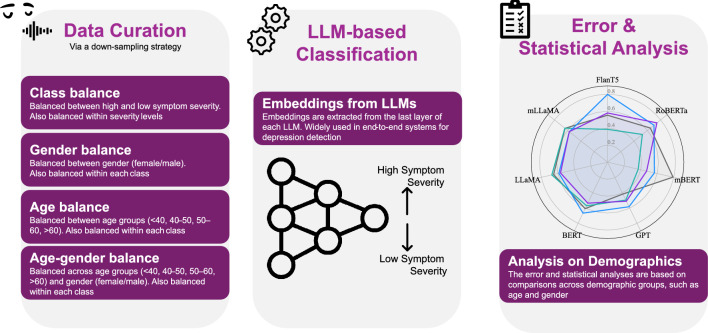
Overview of the experimental pipeline used for evaluating depression detection using LLMs. Left: Data curation ensures balance across class labels (non-balanced), gender, age groups, and age-gender intersections using a down-sampling strategy. Center: LLM-based classification uses final-layer embeddings to detect symptom severity in an end-to-end setup. Right: Error and statistical analysis assess model performance across demographic subgroups, including age and gender, to evaluate potential disparities.

This work explores how demographic biases and dataset imbalances in pre-trained LLMs influence the classification of high depression symptom severity vs. low depression symptom severity. Figure 1 illustrates the workflow followed in this paper. We aim to analyze how age, gender, and their combined biases affect the accuracy of LLM-based models when detecting depression symptom severity. By conducting this analysis across balanced datasets in English (EN), Spanish (ES), Dutch (NL), and a dataset comprising the three languages (All), our experiments will highlight potential demographic biases inherent in these models, and demonstrate their impact on the performance of depression symptom severity classification.

In this study, we define key concepts as follows. Bias refers to systematic differences in model behavior or outputs that correlate with demographic attributes such as age or gender. Fairness denotes equitable performance across demographic groups, typically evaluated by comparing classification metrics. Imbalance describes unequal representation of subgroups in the training data, which may introduce or amplify bias. Performance disparities are observed differences in evaluation outcomes (e.g., F1-score, Unweighted Average Recall–UAR) across demographic or linguistic subgroups. Distinguishing these terms enables a clearer interpretation of results and supports the development of fairness-aware methodologies. To observe disparities in the LLM training data and avoid those arising from the MDD dataset’s lack of balance, we equalized the number of samples (downsampled) in the MDD dataset across demographic groups (severity level, age, and gender), thereby reducing biases caused by the initial uneven distribution. All experiments were conducted with the same amount of data (208 samples) to prevent uneven comparisons arising from sample size differences during training.

For each model, we extracted embeddings from the final hidden layer and fed them into a binary multilayer perceptron classifier with a hidden layer of 256 units. The dataset was split into 70% training, 15% validation, and 15% testing sets, ensuring both category balance and a speaker-independent evaluation protocol. We used the Adam optimizer with an initial learning rate of 1e-3, early stopping (patience = 10), a batch size of 10, and trained for up to 50 epochs.

We conducted five runs of the bootstraping, randomizing the demographic groups with the remaining samples due to balance, and reported the average results. Tab.3 presents the results for balanced demographic differences, where the performance of each model is evaluated using the UAR and F1-score, which provide insight into classification robustness and class-specific performance. The classification task is a binary problem to distinguish between high- and low-symptom severity subjects. Paired t-tests were conducted to assess statistical significance across age, gender, age-gender, and language balancing conditions.

**Table 3:** Comparative analysis of model performance on age- and gender-balanced MDD datasets using different LLMs.

Lang	Model	Non-balanced	Age	Gender	Age-gender
EN	FlanT5	62.78 / 57.87	**75.00 / 71.43**	55.00 / 67.86	53.13 / 61.54
	RoBERTa	61.65 / 60.77	**62.50 / 71.43**	52.50 / 53.66	62.50 / 62.50
	mBERT	56.39 / 59.72	53.13 / 40.00	55.00 / 55.00	**62.50 / 70.00**
	GPT	59.77 / 67.67	**71.88 / 68.97**	47.50 / 61.81	43.75 / 52.63
	BERT	62.03 / 58.43	62.50 / 50.00	**57.50 / 65.31**	59.38 / 62.86
	LLaMA	63.53 / 69.99	**66.52 / 71.91**	54.76 / 29.63	60.71 / 59.26
	mLLaMA	**68.79 / 70.43**	62.50 / 64.71	62.50 / 59.46	65.63 / 62.07
ES	FlanT5	**71.21 / 73.24**	50.00 / 34.42	57.50 / 32.00	53.13 / 28.57
	RoBERTa	**68.18 / 74.70**	46.88 / 45.16	62.50 / 63.41	65.63 / 74.42
	mBERT	65.15 / 56.60	50.00 / 46.67	**67.50 / 74.51**	53.13 / 44.44
	GPT	**75.76 / 77.78**	46.88 / 62.22	60.00 / 60.00	46.88 / 51.43
	BERT	**71.21 / 75.33**	50.00 / 33.33	50.00 / 33.33	53.13 / 66.67
	LLaMA	**65.15 / 68.49**	59.38 / 62.86	65.00 / 58.82	58.38 / 58.07
	mLLaMA	57.14 / 60.44	56.25 / 66.67	60.00 / 50.00	**68.75 / 66.67**
NL	FlanT5	**60.47 / 69.64**	62.50 / 40.00	47.62 / 63.33	53.57 / 38.09
	RoBERTa	60.47 / 64.58	**65.63 / 66.67**	64.29 / 61.54	50.00 / 36.36
	mBERT	59.30 / 59.77	50.00 / 16.67	50.00 / 63.16	**50.00 / 66.67**
	GPT	60.47 / 64.58	46.88 / 58.53	**66.67 / 63.16**	53.57 / 43.47
	BERT	59.30 / 55.70	59.38 / 64.87	**70.05 / 66.67**	50.00 / 30.00
	LLaMA	65.11 / 63.41	56.25 / 61.11	64.27 / 63.42	**66.52 / 68.62**
	mLLaMA	61.65 / 50.00	68.75 / 68.75	**69.05 / 68.29**	62.50 / 62.50
All	FlanT5	**70.33 / 72.07**	61.46 / 59.34	63.71 / 61.54	56.25 / 48.14
	RoBERTa	**66.27 / 65.69**	63.54 / 63.16	63.71 / 57.14	59.38 / 48.00
	mBERT	59.33 / 68.16	**64.58 / 71.67**	62.10 / 69.28	56.25 / 56.25
	GPT	**66.99 / 67.30**	65.63 / 65.26	58.87 / 63.83	51.56 / 52.31
	BERT	**67.22 / 66.99**	64.58 / 65.31	66.13 / 63.79	60.94 / 68.35
	LLaMA	63.88 / 68.21	**67.02 / 66.67**	67.50 / 58.07	64.13 / 62.07
	mLLaMA	**65.31 / 67.56**	62.50 / 66.04	60.48 / 55.05	60.87 / 56.10

The best performance per row (UAR/F1) is bolded.* Lang* Language.

In the case of *non-balanced*, the datasets were only balanced according to different depression severity levels. Regarding *age balance*, the data was divided into four age groups. The age groups were defined as follows: (1) younger than 40 years, (2) between 40 and 50 years, (3) between 50 and 60 years, and (4) older than 60 years. The rationale for this age categorization is that depression manifests differently across life stages, where adolescents, middle-aged adults, and older adults often express distress using distinct linguistic patterns, and their prevalence of depression also varies across these groups^11^. *Gender balance* was achieved by ensuring an equal number of samples in each gender group (women and men) within the MDD classes. Finally, datasets were balanced across *age*, and *gender* simultaneously. For language-specific results, age-balanced datasets considerably improved the performance of FlanT5 (UAR = 75%) and GPT (UAR = 71.88%) models for English in age-balanced datasets. However, age-gender balancing decreased the performance for most models except RoBERTa and mBERT, which performed better with UAR = 62.50%. For Spanish, GPT obtained the best results with the non-balanced dataset (UAR = 75.76%). Age-gender balancing showed varying impacts, with RoBERTa achieving the highest accuracy under this condition (65.63%). Gender balancing seemed to benefit mBERT the most, reaching a 67.50% accuracy. LLaMA and MentaLLaMA showed strong performance in age-balanced settings (UAR up to 67.02%) but were sensitive to age-gender balancing, particularly in English and Spanish. For Dutch, models generally showed moderate performance across all conditions. BERT outperformed in gender-balanced datasets, with a 70.05% of UAR, the highest across this language group. For “All” languages, FlanT5 performed better across non-balanced conditions (UAR=70.33%), followed by BERT with UAR = 67.22%.

Tab. 4 presents the results of the t-tests comparing the performance of the model with different balance strategies. Age-gender balance had the most significant impact on performance, with statistically significant differences (*p*<0.05) observed in nearly all models, including FlanT5, GPT, mBERT, BERT, LLaMA, and mLLaMA. This indicates that jointly controlling for demographic variables meaningfully alters the decision boundaries learned by LLMs. Language variation also had a strong effect on model behavior, with significant differences observed in all models except RoBERTa. However, in some cases RoBERTa show a clear drop in performance while balancing by demographics. highlighting the importance of language-specific adaptation in multilingual mental health tasks. In contrast, gender balance alone did not produce statistically significant changes, suggesting that its effect may be more relevant to fairness than to raw performance. In particular, mBERT showed a significant sensitivity to age balancing, while RoBERTa remained robust under all conditions, showing no statistically significant effects.

**Table 4:** t-Test results comparing model performance across different balancing conditions.

Model	Age		Gender		Age-Gender		Language	
	t	*p*	t	*p*	t	*p*	t	*p*
FlanT5	0.69	0.52	1.67	0.15	**4.36**	**0.00**	**2.67**	**0.01**
RoBERTa	1.17	0.29	1.11	0.31	0.24	0.82	1.46	0.16
mBERT	**4.27**	**0.01**	2.05	0.09	**2.96**	**0.03**	**6.58**	**0.00**
GPT	0.74	0.49	1.38	0.22	**3.75**	**0.01**	**2.69**	**0.01**
BERT	1.40	0.21	0.77	0.47	**2.45**	**0.04**	**2.26**	**0.03**
LLaMA	1.71	0.14	0.79	0.46	**2.56**	**0.04**	**2.46**	**0.02**
mLLaMA	2.04	0.09	1.60	0.16	**3.51**	**0.01**	**3.69**	**0.00**

Statistically significant results (*p* < 0.05) are highlighted in gray and bolded

**Fig. 2:**
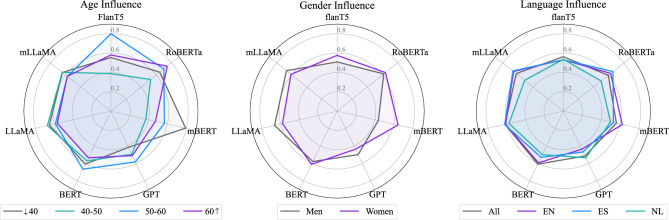
Radar plot illustrating the comparative influence of age, gender, and language on the performance of different LLMs for classifying MDD. Each axis represents a model, and the distance from the center indicates the UAR for each demographic.

By analyzing the age-gender balanced dataset, we observed the impacts of these variables as illustrated in Fig. 2. The radar plot visually underscored these points, illustrating the varying UAR scores across different models when adjusted for demographic groups. This visual analysis supports the quantitative data presented in the tables, offering a clear depiction of how demographic factors influence model accuracy. Each language dataset was balanced to enable a focused evaluation of model performance across diverse demographic groups.

*Age Influence*: The age influence on model performances (Fig. 2) shows a more pronounced variability than gender. Figure 2 highlights mBERT and FlanT5 seem to perform well in distinguishing MDD symptom severity among the youngest age group and the age group between 50 and 60 years old, respectively. This could indicate an age-related language pattern captured effectively by these models. mBERT bias was expected due to the nature of the data on which it was trained (Reddit posts), where most of the participants are more likely composed of a young population^48,49^. GPT’s performance also varies with age but appears more consistent across the age groups than other models.

*Gender Influence*: Impacts on the model performances due to gender variations are noticeable (see Fig. 2). Both BERT and RoBERTa appear relatively equally across genders, while FlanT5, GPT, and mBERT show varying degrees of UARs to gender. We also observe that the differences in performance are not especially pronounced, suggesting that gender, while influential, does not drastically alter the performance of these models in this dataset. Conversely, it appears that mBERT’s performance in gender shows some level of disparity, where there is a noticeable distinction of about 20% in how the model performs when classifying MDD in male vs. female subjects. Interpretation of the results suggests that MDD prevalence might be higher among women^50^. However, the woman population in the training dataset is underrepresented, considering mBERT’s training data came from Reddit, where males predominantly make up 68% of the user base^21^.

*Language Influence*: Language generalization is a clear challenge for the models (Fig. 2), with performance varying across different languages. This suggests that, while models such as FlanT5 and BERT have broad applicability, they require additional fine-tuning to optimize their performance for specific languages. We observed that EN generally showed a higher performance, followed by ES and NL.

## Discussion

This study evaluated the impact of demographic biases on the classification performance of MDD symptom severity using pre-trained LLMs. Through comprehensive analysis across balanced datasets in EN, ES, and NL, as well as a combined dataset of all languages, we demonstrated that age, gender, and their combined balancing affect model performance. To the best of our knowledge, this is the first time such results have been demonstrated in a multilingual context for depression detection applications. These findings highlight the need to understand equality, diversity, and fairness in LLMs, particularly how inherent biases could affect clinical decision-making^51^. Compared to previous work in LLM-based mental health research, our findings both align with and extend the current state of knowledge^7^. Earlier studies have demonstrated that LLMs can detect depression from free text with high accuracy^52^, but they largely focus on monolingual English datasets and seldom report performance across demographic subgroups. Our study advances this by systematically evaluating performance across three languages and explicitly controlling for demographic factors. In particular, our finding that age balancing notably impacts model performance reinforces growing concerns about age-related bias in mental health AI^51^, while our use of multilingual data broadens generalizability beyond most existing work^26^.

Our findings indicate that balancing datasets for specific demographics can influence the performance of models in depression classification. Age balancing appears to have a more pronounced influence on performance, with models such as FlanT5 and GPT exhibiting enhanced accuracy within age-specific datasets. Gender influence, while less pronounced, still showed variability, especially in models trained on datasets with a gender imbalance, such as mBERT, which was trained on predominantly male-generated content from Reddit^43^. LLaMA-based models, including MentalLLaMA, were particularly sensitive to demographic shifts, showing reduced robustness when evaluated on balanced subsets, especially across age groups. At the same time, age-gender balance condition provided mixed results, suggesting that the interaction between these factors and model performance might be complex and model-dependent. While LLMs often achieve F1-scores above 0.90 on social media data^14^, our results on multilingual speech transcripts (F1: 0.50–0.73%, UAR: 0.53–0.75%) align with existing benchmarks in spoken depression detection, where performance remains more modest due to ASR noise and linguistic variability^16^.

The analysis of language generalization capabilities highlighted differences in model performances across languages. Certain models outperformed in specific languages but showed varied results in the combined dataset. For instance, models generally achieved higher accuracy in English, likely due to the richer and more comprehensive training data available in this language compared to Spanish and Dutch. Note that the majority of datasets used to train these LLMs are predominantly in English and lack comprehensive metadata (see Tab. 2). Furthermore, many of these corpora are derived from webpages, complicating the clarity of data provenance. Additionally, most sources originate from formal written language, such as Wikipedia, rather than spoken language. Given that communication capabilities are often impacted in mental disorders such as depression, the predominance of formal language training in these models represents a significant limitation.

We aimed to achieve equal representation across various age groups, genders, and severity levels by controlling sample sizes. Note that no current clinical depression dataset achieves perfect sociodemographic balance, and biases related to MDD data can still be present in this analysis despite the extensive demographic balancing performed in this study.

While we strived to control for age and gender in our dataset, several limitations should be acknowledged. Although sociodemographic fairness is a broader issue, our analysis was restricted to age and gender because including additional attributes would have significantly reduced the usable dataset after undersampling. As a result, important factors such as ethnicity, education, and socioeconomic status, known to influence mental health outcomes and could not be analyzed here. Future work should consider how to integrate these attributes through more scalable balancing methods or by leveraging larger, more diverse datasets. Moreover, despite our efforts to demographically balance, residual biases from the pretraining data, typically scraped from English-dominant web-based sources, are likely to persist. External validation was also not feasible due to the scarcity of multilingual depression datasets with standardized protocols. Finally, the specificity of our clinical use case limits generalizability. While our results point to meaningful trends, especially regarding age effects on model performance, broader validation is essential to confirm whether these findings hold in other contexts.

## Conclusion

This study presents a systematic evaluation of demographic bias in pre-trained LLMs for multilingual depression detection using speech-derived text. By analyzing data sets in English, Spanish, and Dutch^26^, and applying controlled balance between age, sex, and their intersection, we demonstrate that demographic factors, particularly age, significantly influence model performance^43,51^. Combined age-gender balance and language variation also produce notable shifts in classification outcomes, underscoring the complexity of fairness in clinical NLP tasks ^51^. Our comparative analysis includes both general-purpose and mental health-tuned LLMs^43^, evaluated using UAR and F1-score to ensure robust, subgroup-sensitive assessment^53^. These findings reinforce the importance of demographic-aware design in clinical AI pipelines and support the need for fairness evaluation protocols in mental health applications^24^. Applications of LLMs in mental health screening must contend with biases that stem both from training data and population sampling. Our study highlights the importance of demographic balancing for evaluating model performance across age and gender groups. While balancing via downsampling allowed for controlled comparisons, it also constrained dataset size and limited the inclusion of other key attributes such as ethnicity, education, or socioeconomic status. To move beyond these limitations, future work should explore algorithmic bias mitigation techniques such as adversarial training, which can enforce invariance to demographic attributes during optimization, and contrastive learning^53^, which encourages models to learn demographically robust representations. Since transcription quality can influence downstream model behavior, especially in multilingual settings, it is also important to account for potential biases introduced during automatic speech recognition. Although Whisper (large-v3) demonstrates strong performance across languages, its transcription accuracy may still vary by language and speech characteristics, which could propagate inequities in classification outcomes. Finally, external validation on larger, culturally diverse datasets is essential to ensure the broader applicability and clinical robustness of LLM-based depression screening tools. This includes evaluation across varying socio-demographic profiles, languages, and healthcare contexts, as well as the use of both elicited and spontaneous speech data (considered in this work). Such validation will help determine the generalizability of model performance and fairness across unseen populations and real-world deployment settings, especially in the presence of noisy transcriptions and cross-linguistic variability.

## Data Availability

Due to the sensitive nature of the clinical speech data and ethical requirements to protect participant confidentiality, the dataset used in this study cannot be made publicly available. However, access to the data may be granted upon reasonable request and subject to local ethics clearances through the RADAR-CNS consortium. Requests for access can be directed to the senior author at nick.cummins@kcl.ac.uk.

## Code availability

The code used for data preprocessing, model training, and demographic balancing will be made publicly available at https://github.com/PauPerezT/LLM-BIAS-MD.
